# Are Proteinase 3 and Cathepsin C Enzymes Related to Pathogenesis of Periodontitis?

**DOI:** 10.1155/2014/420830

**Published:** 2014-05-19

**Authors:** Oya Türkoğlu, Elif Azarsız, Gülnur Emingil, Necil Kütükçüler, Gül Atilla

**Affiliations:** ^1^Department of Periodontology, School of Dentistry, Ege University, 35100 Izmir, Turkey; ^2^Department of Pediatric Immunology, School of Medicine, Ege University, 35100 Izmir, Turkey

## Abstract

*Aim*. Cathepsin C is the activator of the polymorphonuclear leukocyte-derived proteinase 3, which contributes to inflammatory processes. The aim of the present study was to investigate gingival crevicular fluid (GCF) proteinase 3 and cathepsin C levels in periodontal diseases. *Design*. Eighteen patients with chronic periodontitis (CP), 20 patients with generalized aggressive periodontitis (G-AgP), 20 patients with gingivitis, and 18 healthy subjects were included in the study. Periodontal parameters including probing depth, clinical attachment level, papilla bleeding index, and plaque index were assessed in all study subjects. GCF proteinase 3 and cathepsin C levels were analyzed by ELISA. *Results*. GCF proteinase 3 total amount was significantly higher in diseased groups compared to control group, after adjusting age (*P* < 0.05). No differences were found in GCF cathepsin C levels among the study groups (*P* > 0.05). Periodontal parameters of sampling sites were positively correlated with GCF proteinase 3 total amounts (*P* < 0.01) but not with cathepsin C total amounts (*P* > 0.05). *Conclusions*. Elevated levels of GCF proteinase 3 in CP, G-AgP, and gingivitis might suggest that proteinase 3 plays a role during inflammatory periodontal events in host response. However, cathepsin C in GCF does not seem to have an effect on the pathogenesis of periodontal diseases.

## 1. Introduction

Periodontitis is a disease caused by a bacterial infection leading to gingival inflammation, destruction of periodontal tissues, and tooth loss. Tissue destruction depends on the balance between host protective and destructive mechanisms [1]. Since periodontal tissues are constantly exposed to a variety of microbial pathogens the innate immune response has a primary role in the defense against plaque-associated bacteria in this complex environment [2, 3].

Neutrophils are the primary cells recruiting the inflammation site in response to a microbial challenge and they are important contributors to the innate immune [4, 5]. They produce antimicrobial peptides including defensins, human neutrophil peptides (HNP), and cathelicidins that contribute to maintaining the balance between health and disease [2]. By expressing cathelicidin antimicrobial peptides neutrophils amplify innate immune responses against invading pathogens [6]. Human cathelicidin antimicrobial peptide of 18 kDa (hCAP18) consists of a cathelin-like domain and a C-terminal peptide called LL-37 [7]. After inflammatory stimulus hCAP18 is cleaved to LL-37 and cathelin-like domain by proteinase 3 [8]. Proteinase 3 is a 29 kDa serine proteinase that is stored in azurophilic granules of polymorphonuclear neutrophils (PMNs) [9]. Exposure of PMNs to cytokines or chemoattractants gives rise to an increase in cells surface-bound proteinase 3 [10]. Proteinase 3 has many functions, such as degradation of extracellular matrix proteins [11], platelet activation [12], induction of apoptosis [13], and increase of TNF-*α* and IL-1*β* release [14]. Proteinase 3 and antimicrobial peptides are involved in nonoxidative killing of microorganisms [15, 16]. Proteinase 3 actively contributes to inflammatory processes [17] and has a crucial function in the regulation of innate immune responses against microorganisms [18, 19].

Cathepsin C plays essential role in the host defense against bacteria [18] and is the activator of the PMN-derived serine proteinases such as elastase and proteinase 3 in human [19]. The absence of cathepsin C is found to be related to a diminished activity of the PMN-derived proteases in patients with Papillon-Lefevre syndrome (PLS) [19–21]. PMN's capacity to neutralize leukotoxin and eliminate* Aggregatibacter actinomycetemcomitans* seems to be reduced in patients with reduced cathepsin C activity [22]. Additionally the importance of cathepsin C in human natural killer cell function in infection control has been emphasized in different studies [20, 21, 23, 24]. It has been stated that impaired killer cell cytotoxicity might contribute to the pathogenesis of PLS-associated periodontitis [23, 24]. Mutations in the cathepsin C gene have been identified in prepubertal aggressive periodontitis [25–27]. It has been suggested that cathepsin C gene variants contribute to increased susceptibility in generalized aggressive periodontitis [28].

There are limited numbers of studies investigating the roles of proteinase 3 and cathepsin C in the pathogenesis of periodontal diseases [29–31]. Komine et al. have demonstrated that the detection ratio of the proteinase 3-like activity is elevated in the parotid saliva of periodontitis patients [30]. Laugisch et al. have showed that the highest activity of GCF proteinase 3 is detected in gingivitis patients, followed by chronic periodontitis and aggressive periodontitis patients [31]. Researchers have also suggested that periodontopathogenic bacteria stimulate the release of proteinase 3 [31]. Cathepsin C activity has been found to be significantly lower in tissue extract supernatants and gingival crevicular fluid (GCF) of periodontitis patients compared with those of healthy controls [29]. We have previously reported that local deficiency in hCAP-18/LL-37 and p.S34N mutation in CAMP gene, which is the gene encoding the LL-37, might be a confounding factor in the pathogenesis of generalized aggressive periodontitis [32, 33]. Therefore, in the present study it was hypothesized that hCAP-18/LL-37 deficiency might be caused by the lack of proteinase 3 and cathepsin C enzymes in GCF of patients with generalized aggressive periodontitis, and the deficiency of these enzymes might contribute to multifactorial etiology of generalized aggressive periodontitis, by modifying host responses. In order to test this hypothesis, we aimed to determine the GCF levels of proteinase 3 and cathepsin C in patients with different periodontal diseases.

## 2. Material and Methods

A total of 76 subjects were included in the present study: 18 chronic periodontitis, 20 generalized aggressive periodontitis, 20 gingivitis patients, and 18 healthy subjects. All subjects were recruited from the Department of Periodontology, School of Dentistry, Ege University. The purpose and procedures were fully explained to all subjects prior to participation, and all participants gave written informed consent in accordance with Helsinki Declaration. The study protocol was approved by the Ethics Committee of the School of Medicine, Ege University (number 12-2.1/4). Patients with systemic diseases such as diabetes mellitus, immunologic disorders, hepatitis, and human immunodeficiency virus infections were excluded, as were pregnant and lactating women and those taking oral contraceptive drugs. None of the subjects received antibiotics within the previous 3 months or treatment for periodontal disease within the last 6 months prior to the study. The dentition of each volunteer was examined clinically and radiographically to assess the suitability of the subjects for the study.

### 2.1. Clinical Periodontal Parameters

The probing depth (PD), clinical attachment level (CAL), plaque index (PI) [34], and bleeding on probing (BOP) [35] were determined at six sites per tooth in the whole mouth, excluding third molars. The papilla bleeding index (PBI) was also assessed [36]. A manual William's periodontal probe (Hu-Friedy, Chicago, IL) was used for PD (millimeters) and CAL (millimeters) measurements. All measurements were performed by a calibrated examiner (OT). The intraexaminer reliability was high as revealed by an intraclass correlation coefficient of 0.87 and 0.85 for PD and CAL measurements, respectively.

### 2.2. Study Groups

Study groups were classified into the four groups based on their periodontal conditions according to criteria proposed by the 1999 International World Workshop for a Classification of Periodontal Disease and Conditions [37].

*Healthy Control.* A total of 18 systemically and periodontally healthy subjects were included in the healthy control group. Healthy subjects had no sites with PD > 3 mm and CAL > 2 mm, a BOP score of <15% at the examination, and no alveolar bone loss was observed (i.e., the distance between the cementoenamel junction and bone crest ≤ 3 mm at >95% of the proximal tooth sites). 

*Gingivitis.* Twenty patients were included in the gingivitis group. A diagnosis of gingivitis was based on the presence of BOP at >50% of sites in the whole mouth of each patient. Patients had no clinical signs of periodontitis and no radiographic evidence of alveolar bone loss (i.e., the distance between the cementoenamel junction and bone crest ≤ 3 mm at >95% of the proximal tooth sites). 

*Chronic Periodontitis.* The chronic periodontitis group included 18 patients with severe generalized chronic periodontitis. They had at least four nonadjacent teeth with sites with CAL > 4 mm and PD > 5 mm, and the CAL was commensurate with the amount of plaque accumulation on the teeth. BOP was >50% for the whole mouth in all patients. 

*Generalized Aggressive Periodontitis.* The generalized aggressive periodontitis group included 20 patients with 16 teeth at least. The patients demonstrated a generalized pattern of severe destruction and CAL > 4 mm and PD > 5 mm on eight or more teeth; at least three of these were other than central incisors of first molars. The extent and severity of bone support and/or osseous lesions were evaluated radiographically in each patient.

### 2.3. GCF Sampling

Study subjects were recalled for GCF sampling a day after the periodontal examination. Sampling sites were selected from buccal aspects of the mesial/distal surfaces at the interproximal sites of single-rooted teeth. GCF samples from patients with chronic periodontitis and generalized aggressive periodontitis were collected from one site of a tooth exhibiting PD > 5 mm and CAL > 4 mm. In patients with gingivitis, GCF samples were obtained from one site of a tooth with BOP and without clinical attachment loss. In the healthy group, GCF samples were collected from one site of a tooth exhibiting PD < 3 mm without clinical attachment loss and BOP. First, supragingival plaque was removed from the interproximal surfaces and surfaces were gently dried and isolated by cotton rolls before GCF sampling. GCF was sampled with paper strips (Periopaper, ProFlow, Inc., Amityville, NY). Paper strips were inserted 1 mm into the crevice and left for 30 seconds. Strips contaminated with blood were discarded. The absorbed GCF volume of each strip was determined by a calibrated electronic device (Periotron 8000, ProFlow, Inc., Amityville, NY) and placed into a sterile polypropylene tube and kept at −80°C. The readings from this electronic device were converted to an actual volume (microliters) by reference to the standard curve.

### 2.4. GCF Proteinase 3 and Cathepsin C Analyses

GCF samples were analyzed by enzyme-linked immunosorbent assay (ELISA). Before the quantitation of proteinase 3 and cathepsin C, GCF samples were eluted from the strips by placing them in 2 mL phosphate buffered saline by shaking the tubes on an ELISA plate shaker for 45 minutes. GCF proteinase 3 and cathepsin C levels were assayed with commercially available ELISA kits (Human Proteinase 3, Uscn Life Science Inc., Wuhan, China) (Human Cathepsin C, Uscn Life Science Inc., Wuhan, China) according to the manufacturers' instructions. The minimum detectable limits of proteinase 3 and cathepsin C were 0.068 ng/mL and 0.113 ng/mL, respectively. The levels of proteinase 3 and cathepsin C in each sample were calculated based on the dilution, and the results were expressed as the total amount and concentration in GCF sample. A calculation of the concentration data for each enzyme was performed by dividing the amount of each mediator by the volume of the sample.

### 2.5. Statistical Analyses

Post hoc power calculations were performed at the 0.05 significance level. Power was calculated as 0.97 for the total amount of proteinase 3. All data analyses were performed using a statistical package (SPSS Inc., ver. 17.0, Chicago, IL, USA). Parametric and nonparametrical techniques were used for statistical analyses. One-way analysis of variance tests were used for intergroup comparisons of age; when there were significant differences (*P* < 0.05), post hoc two-group comparisons were performed with the Dunnett *C* test. The Chi-square test was used for gender comparisons among the study groups. Comparisons of clinical periodontal parameters for whole mouth and sampling sites were made by analysis of one-way analysis of variance or Mann-Whitney *U* tests as appropriate, and post hoc two-group comparisons were assessed by the Dunnett *C* or Bonferroni tests. The Kolmogorov-Smirnov normality test was applied to examine the distribution normality of the data for GCF proteinase 3 and cathepsin C levels. GCF proteinase 3 levels were compared using one-way analysis of variance and post hoc two-group comparisons were assessed by the Bonferroni test. Then the univariate analysis of variance was used to compare the differences in GCF proteinase 3 levels among the study groups after adjusting for age. Kruskal-Wallis test was used to compare the GCF cathepsin C levels among the study groups. The same analysis was applied to compare the GCF cathepsin C levels among the study groups after adjusting for age. Pearson correlation analyses were used to analyze the correlations between GCF proteinase 3 and cathepsin C levels and clinical periodontal parameters.

## 3. Results

### 3.1. Clinical Findings

The demographic data of the study groups are shown in Table 1. There were no significant differences in gender among the study groups (*P* > 0.05). The patients in chronic periodontitis group were significantly older compared to patients in other groups (*P* < 0.05).

Table 2 shows the whole mouth clinical periodontal parameters of the study groups. As expected the whole mouth mean PD, PBI, BOP, and PI scores in the diseased groups were significantly higher compared to those of healthy control group (*P* < 0.05). There were no significant differences in whole mouth periodontal parameters between chronic periodontitis and generalized aggressive periodontitis patients (*P* > 0.05). The whole mouth PD and CAL values of periodontitis patients were significantly higher than those of gingivitis patients (*P* < 0.05). Patients with generalized aggressive periodontitis had significantly lower whole mouth PBI and plaque accumulation percentage compared to gingivitis patients (*P* < 0.05). Clinical periodontal parameters of the sampling sites in study groups are also demonstrated in Table 2. PD and PI scores and GCF volumes of sampling sites were significantly higher in diseased groups than those of healthy controls (*P* < 0.05) and PI scores of sampling sites in periodontitis groups were significantly higher compared to those of gingivitis patients (*P* < 0.05). Sampling sites of chronic periodontitis patients had significantly higher PI score than those of generalized aggressive periodontitis patients (*P* < 0.05) though there were no significant differences in PD, CAL, PBI scores, or GCF volumes between two periodontitis groups (*P* > 0.05).

### 3.2. Proteinase 3 and Cathepsin C Levels

Table 3 shows the total amount of GCF proteinase 3 in the study groups. After adjusting for age, periodontitis and gingivitis groups had significantly higher GCF proteinase 3 total amounts compared to healthy control group (*P* < 0.05). Generalized aggressive and chronic periodontitis groups had similar total amount of GCF proteinase 3 (*P* > 0.05) and there was no significant difference in the total amount of GCF proteinase 3 between gingivitis and periodontitis groups after adjusting for age (*P* > 0.05) (Table 3). GCF proteinase 3 concentrations in the study groups are shown in Table 3. There was no significant difference in the GCF proteinase 3 concentrations between patients with periodontal diseases and healthy controls, after adjusting for age (*P* > 0.05) (Table 3). The distribution of the total amount and concentration of GCF cathepsin C of the study groups are given in Table 3. All study groups had similar total amount and concentration of GCF cathepsin C after adjusting for age (*P* > 0.05) (Table 3).

Correlations between clinical periodontal parameters of sampling sites and GCF proteinase 3 and cathepsin C levels are demonstrated in Table 4. Pearson correlation analysis revealed significant correlations between GCF proteinase 3 total amounts and clinical periodontal parameters of sampling sites (*P* < 0.01). Correlation analysis also revealed positive correlations between GCF proteinase 3 total amounts and cathepsin C total amounts (*P* < 0.01). However no significant correlations were found in clinical periodontal parameters of sampling sites and cathepsin C levels except the correlation between GCF volume of sampling sites and cathepsin C total amount (*P* > 0.01) (Table 4).

## 4. Discussion

Neutrophils are important contributors to innate immune system because they are phagocytic cells protecting the host against bacterial infection and invasion [4]. Besides, neutrophil clearance from the inflammation site in time is also essential for resolution of inflammation because neutrophils might cause tissue damage due to their matrix-degrading enzymes [4, 38]. Neutrophil-derived molecules such as proteinase 3 and cathepsin C participate in inflammation during host response [18, 31]. The present study demonstrated that proteinase 3 total amounts were significantly higher in periodontitis and gingivitis groups than healthy controls but not significantly different among the diseased groups. Additionally, there were positive correlations between GCF proteinase 3 total amounts and clinical periodontal parameters of sampling sites. However, the results of the present study did not reveal significant differences in GCF cathepsin C levels among the study groups. No correlation was found between GCF cathepsin C total amount and clinical periodontal parameters of sampling sites.

Cathepsin C is the enzyme whose activity decreases in periodontitis [29]. Mutations in cathepsin C gene were found to be related to prepubertal periodontitis [25, 27]. Noack et al. stated that cathepsin C gene variants contribute to increased susceptibility in generalized aggressive periodontitis [28]. However, heterozygous family members showing a reduced cathepsin C activity were not affected by periodontitis [28]. It was suggested that very small activity of cathepsin C would be enough for being protected from periodontal infections [39]. Hewitt et al. did not find significant difference in cathepsin C activity between patients with aggressive periodontitis and healthy controls [26]. Soell et al. investigated the activity of cathepsin C in tissue extract supernatants and GCF samples of patients with periodontitis and healthy controls [29]. Researchers stated that cathepsin C activity was significantly lower in diseased samples compared to healthy samples for GCF, and cathepsin C might be involved in adult periodontitis patients not suffering from PLS [29]. There is cumulative evidence that neutrophils in periodontitis are “primed” state to release increased levels of cytokines and matrix-degrading enzymes [4, 40, 41]. However, increased levels of neutrophil-derived enzymes would not exactly reflect that neutrophils express effectively working enzymes. In the present study we failed to show decreased levels of GCF cathepsin C in diseased groups compared with healthy controls. That might be explained by the fact that primed neutrophils in periodontitis secrete their matrix-degrading enzymes into the periodontal tissues but secreting enzyme levels were not found to be different among the diseased groups because “priming” neutrophil would not mean “effective” working neutrophil. The activity of cathepsin C enzyme in the study groups was not evaluated in the present study. To the best of our knowledge, there is no study evaluating GCF cathepsin C levels in different types of periodontal diseases. Therefore, studies investigating the activity together with the levels of cathepsin C enzyme in generalized aggressive and chronic types of periodontitis and gingivitis would be useful for analyzing the role of this enzyme in the pathogenesis of periodontal diseases.

Holzhausen et al. stated that patients with chronic periodontitis presented significantly higher levels of proteinase 3 mRNA expression in GCF than healthy controls [42]. In the present study GCF proteinase 3 levels were significantly higher in both periodontitis and gingivitis groups compared to healthy controls. The higher levels of GCF proteinase 3 levels in diseased groups might be caused by the presence of increased numbers of neutrophils in the inflammatory area. Defective clearance of neutrophils from the periodontal tissues would predispose to nonhealing chronic inflammation [4].* Porphyromonas gingivalis* (*P. gingivalis*) belongs to red complex within subgingival biofilms, and it is highly correlated with gingival inflammation and periodontal destruction [43].* P. gingivalis* proteases are able to degrade protease inhibitors effectively [44]. Therefore, increased levels of GCF proteinase 3 might also be caused by the degradation of protease inhibitors by* P. gingivalis*. Laugisch et al. suggested that periodontopathogenic bacteria stimulated the release of proteinase 3, and the highest activity of proteinase 3 was detected in gingivitis patients, followed by periodontitis patients [31]. Studies evaluating proteinase inhibitors together with proteinase 3 in periodontitis and gingivitis would provide information about the role of this enzyme in the periodontal pathogenesis.

In the present study, it is demonstrated that there were significant correlations between GCF proteinase 3 total amounts and clinical periodontal parameters of sampling sites such as PD, CAL, PI, and PBI. Increasing microbial plaque amounts stimulate inflammatory and immune responses during progression of periodontal disease. As the probing depth increases it creates an anaerobic environment, and the anaerobic conditions in periodontium make the neutrophils express more compounds independent of oxygen, such as antimicrobial peptides and proteinases [22, 45, 46]. Therefore, it is not surprising to find positive correlations between proteinase 3 total amounts and periodontal parameters of sampling sites in the present study. Additionally, positive correlations were demonstrated between GCF proteinase 3 and cathepsin C total amounts in the present study. This correlation is not surprising when considering the role of cathepsin C in the activation of PMN-derived serine proteinases elastase, cathepsin G, and proteinase 3.

Serine proteinases, such as proteinase 3, play important role in the processing of hCAP18 into hCAP18/LL-37 antimicrobial peptide [8]. It is known that proteinase 3 together with antimicrobial peptides hCAP18/LL-37 forms the basis of the oxygen-independent killing mechanism of PMNs [46, 47]. In the present study, no significant difference was found in GCF proteinase 3 levels among the diseased groups. Our previous results demonstrated that the level of GCF HNP1-3 was not different among the patients with gingivitis, generalized aggressive periodontitis, and chronic periodontitis [48]. Besides, we reported that chronic periodontitis patients had significantly higher GCF LL-37 levels compared to healthy controls [49]. hCAP18 is stored in the secondary granules of neutrophils, while proteinase 3 and HNP1-3 were stored in azurophilic granules [7, 9, 50]. Taken together it might be suggested that defects in or the secretion of azurophilic granules of neutrophils is partly responsible for not significantly elevated levels of GCF proteinase 3 in periodontitis groups more than in gingivitis groups.

The cross-sectional study design prevents us from determining the exact nature of the increased GCF levels of proteinase 3 and unchanged levels of GCF cathepsin C. The GCF samples were collected in only one time point in the present study. Although the sampling sites had periodontal inflammation during GCF sampling, it could not be suggested that the sampling sites had progressing periodontitis in that time point. Another limitation of the present study is that GCF hCAP18/LL-37 levels were not evaluated in the same GCF samples in which proteinase 3 and cathepsin C levels were analyzed in order to test our hypothesis.* In vitro* studies searching the effect of these enzymes on each other and hCAP18 in neutrophils obtained from the patients with different periodontal diseases would supply important data for enlightening the possible deficiencies in neutrophils of these patients. Despite these limitations, a significant relationship between periodontal diseases and GCF levels of proteinase 3 was observed in the present study.

## 5. Conclusion

Our results demonstrated that GCF proteinase 3 total amounts in periodontitis and gingivitis groups were significantly higher compared to healthy control but similar among the diseased groups. Additionally, there were positive correlations between GCF proteinase 3 total amounts and clinical periodontal parameters of sampling sites. The data of the present study might suggest a role for proteinase 3 during inflammatory immune responses in the pathogenesis of periodontal disease. Our results revealed no significant differences in GCF cathepsin C levels among the study groups. Further studies are needed to evaluate the activity of cathepsin C and proteinase 3 enzymes in different periodontal diseases as well as after periodontal treatment. Additionally,* in vitro* studies analyzing the enzyme levels and effectiveness of secreting from different granules of primed state neutrophils obtained from patients with periodontitis would provide important data for pathogenesis of periodontal diseases.

## Figures and Tables

**Table 1:** The demographic data of the study groups.

	Healthy (*N* = 18)	Gingivitis (*N* = 20)	CP (*N* = 18)	G-AgP (*N* = 20)
Age	31 ± 9.5	32 ± 10	46 ± 9*	29 ± 4
Gender (F/M)	11/7	13/7	11/7	12/8

*Significant difference from other groups.

CP: chronic periodontitis; G-AgP: generalized aggressive periodontitis; F: female; M: male.

**Table 2:** Clinical periodontal parameters of the study groups (mean ± SD).

	Healthy (*N* = 18)	Gingivitis (*N* = 20)	CP (*N* = 18)	G-AgP (*N* = 20)
Whole mouth				
PD (mm)	1.71 ± 0.14*	2.17 ± 0.25^†^	3.69 ± 0.66	3.73 ± 0.9
CAL (mm)	0.05 ± 0.04^†^	0.05 ± 0.01^†^	4.64 ± 1.18	4.4 ± 1.3
PBI	0.13 ± 0.06*	2.62 ± 0.29^‡^	2.35 ± 0.5	2.08 ± 0.66
PI	1.4 ± 0.26*	3.3 ± 0.45^‡^	3.07 ± 0.42	2.59 ± 0.78
BOP (%)	6 ± 2.5*	82 ± 10	83 ± 13	76 ± 19
PI (%)	23 ± 12*	98 ± 2^‡^	93 ± 9	84 ± 13
Sampling sites				
PD (mm)	2 ± 0.6*	2.75 ± 0.85^†^	7.2 ± 1.6	6.9 ± 1.8
CAL (mm)	0	0	7.3 ± 2.1	7.1 ± 2.1
PBI	0	2.45 ± 0.5^§^	3 ± 0.8	2.6 ± 0.9
PI	0.8 ± 0.5*	3.3 ± 0.6^†^	3.8 ± 0.5^‡^	2.5 ± 1.2
GCF (*μ*L)	0.17 ± 0.1*	0.36 ± 0.15	0.39 ± 0.18	0.34 ± 0.18

*Significant difference from other groups.

^†^Significant difference from CP and G-AgP.

^‡^Significant difference from G-AgP.

^§^Significant difference from CP.

CP: chronic periodontitis; G-AgP: generalized aggressive periodontitis; PD: probing depth; CAL: clinical attachment level; PBI: papilla bleeding index; PI: plaque index; BOP: bleeding on probing; GCF: gingival crevicular fluid; SD: standard deviation.

**Table 3:** GCF proteinase 3 and cathepsin C levels in sampling sites of the study groups (estimated mean ± SE [95% CI]).

	Healthy (*N* = 18)	Gingivitis (*N* = 20)	CP (*N* = 18)	G-AgP (*N* = 20)
PR3 (ng/sample)	1.91 ± 0.23*	2.86 ± 0.2	3.22 ± 0.28	3.22 ± 0.2
PR3 (ng/*μ*L)	14.39 ± 1.8	10.41 ± 1.7	9.02 ± 2.11	11.49 ± 1.7
CTSC (ng/sample)	0.25 ± 0.30	0.81 ± 1.2	0.3 ± 0.25	0.8 ± 0.97
CTSC (ng/*μ*L)	2.34 ± 4.02	2.21 ± 2.65	0.8 ± 0.67	2.45 ± 2.77

*Significant difference from other groups.

CP: chronic periodontitis; G-AgP: generalized aggressive periodontitis; PR3: proteinase 3; CTSC: cathepsin C; SE: standard error.

**Table 4:** Correlations between proteinase 3 (PR3) and cathepsin C (CTSC) levels and clinical periodontal parameters in the study groups.

	PR3 (ng/sample)	PR3 (ng/*μ*L)	CTSC (ng/sample)	CTSC (ng/*μ*L)
PD (mm)	0.534*	−0.117	0.145	−0.040
CAL (mm)	0.377*	−0.104	0.083	−0.042
PI	0.407*	−0.306*	0.222	−0.017
PBI	0.528*	−0.169	0.221	0.000
GCF (volume)	0.417*	−0.585*	0.325*	−0.127
CTSC (ng/sample)	0.442*	−0.092	1	0.730*

*Correlation is significant at the 0.01 level (2-tailed).

PD: probing depth; CAL: clinical attachment level; PI: plaque index; PBI: papilla bleeding index; GCF: gingival crevicular fluid.
